# Being a Swedish university student in a country far away: a qualitative study

**DOI:** 10.1186/s12889-025-25929-6

**Published:** 2025-12-12

**Authors:** Emil Danehorn, Goldina Smirthwaite, Marie Oscarsson, Ulla Peterson, Katarina Swahnberg

**Affiliations:** 1 Faculty of Health and Life Sciences, Linnaeus University, Kalmar, Sweden; 2 Faculty of Arts and Social Sciences, Karlstad University, Karlstad, Sweden

**Keywords:** Swedish exchange students, Stress, Precautions, Sex, Alcohol, Violence

## Abstract

**Background:**

In Sweden, during the school years of 2021/22, 11,900 women and 8,100 men chose to spend a semester abroad; however, there has been little research on how they experience their time abroad and what difficulties they might face. Some studies have shown that Swedish exchange students consume alcohol to a higher degree and indulge in risky sexually behaviour while on exchange studies. Therefore, our aim was to explore Swedish exchange students’ experiences from a semester abroad.

**Methods:**

A qualitative design with semi-structured interviews. Eleven students who had spent parts of their education abroad participated in the study. A qualitative content analysis was used to analyse the data.

**Results:**

The exchange students experienced stress due to a high workload and found that cultural differences could be both frustrating and fascinating. Some exchange students experienced minor illnesses and homesickness. The exchange students expressed a responsible view on sex and emphasized using protection against STIs while engaging in casual sex. Some enjoyed drinking alcohol to varying degrees and meeting new friends while abroad. However, they rarely engaged in excessive drinking; instead, they adopted a more spontaneous and social drinking pattern. Some experienced violence, such as robbery and harassment, and most took several safety measures, including being extra careful to lock their doors, not going out alone, and using public transport instead of taxis.

**Conclusion:**

Swedish exchange students described stress and casual alcohol consumption as common experiences. They emphasized the importance of STI protection as well as safety measures to avoid exposure to violence. Reports of alcohol consumption, casual sex, violence, minor illnesses, and homesickness highlight the challenges faced during exchange studies. These findings indicate potential risks associated with being an exchange student.

**Supplementary Information:**

The online version contains supplementary material available at 10.1186/s12889-025-25929-6.

## Background

In Sweden, during the school years of 2021/22, 11,900 women and 8,100 men chose to spend a semester abroad. A majority of the students were between 22 and 24 years of age [1]. There are several reasons why Swedish students travel abroad, such as cultural experiences, the opportunity to visit other countries, and personal growth. However, it also requires courage and a willingness to take risks [2]. Exchange studies, for Swedish students, are also associated with high personal financial costs, even if the students have received some kind of scholarship and there are no tuition fees [3].

International studies on exchange students showed that while abroad, many exchange students prioritize being a tourist, engage in social activities before studies, as well as travelling if they can afford it ^4^. However, exchange programmes can also help students identifying important life skills, such as developing new interests, as well as boost their self-efficiency and self-confidence [4]. There are also several practical benefits of being an exchange student, mainly connected to job opportunities, such as developing communications skills [5]. However, research indicates that exchange students often increase their alcohol consumption while abroad [6–8], sometimes to levels exceeding those of the local students [8]. Furthermore, it has been shown that exchange students tend to prioritize social activities and partying with friends over studying [9]. Research has also shown sexually risky behaviour among female exchange students in the US, where 17% engaged in sexual activity with a new male partner during their exchange studies, but only 23% used a condom regularly [10]. Exchange students have also described a stigma associated with carrying condoms, as this behavior could be interpreted as promiscuous, and reported that condoms were perceived to reduce sexual pleasure [11].

Even before embarking on a semester abroad, Swedish students who intend to do so (referred to as prospective exchange students), exhibit differences compared to students who do not plan to travel (referred to as campus students). Prospective exchange students tend to consume more alcohol during a single occasion, engage in sexual activity with new partners, and have sex under the influence of alcohol to a higher degree than campus students [12]. Additionally, there are notable differences between sexes: male prospective exchange students are more likely to have sex under the influence of alcohol compared to male campus students, while female prospective exchange students are more likely to have sex with more partners and drink alcohol more often than their female campus student counterparts [12]. On the other hand, it has been shown that risky alcohol consumption among Swedish youths between 17 and 27 has been steadily decreasing in recent years [13]; however, the decrease is not as prominent for heavy drinkers as for youths who drink less [14]. Swedish prospective exchange students also report better mental health than campus students, and male participants in both groups reported better mental health than their female counterparts [12].

During their semester abroad, Swedish exchange students tend to increase their weekly consumption of alcohol, have more sex with new partners than campus students, and have sex with more than three partners [15]. They also expose themselves to sexual risks, such as sex without a condom. Furthermore, it has been shown that one-sixth of Swedish exchange students had been subjected to sexual violence while abroad [16]. Swedish exchange students also rate their mental health as being better than that of campus students [15].

Research indicates that Swedish exchange students already differ from regular students before their semester abroad; they indulge in activities such as a high alcohol consumption and have sex under the influence of alcohol. During their exchange trip, they increase their frequency of alcohol consumption and expose themselves to sexual risks, such as sex without a condom, which could potentially affect their well-being. There are also reports that Swedish exchange students have been exposed sexual violence while abroad.

There has been limited research on Swedish exchange students and their experiences from abroad. To increase the understanding of health-related behaviours among Swedish exchange students and to develop the interview guide for this study, we conducted two quantitative studies investigating sexually risky behaviour, alcohol use, drug use, mental health, and self-rated health, both before [12] and after a semester abroad [15]. By exploring former exchange students’ experiences from abroad, health-promoting interventions could be developed, for example, by using the Diffusion of Innovation model [17]. It is a model that explains the spread of health behaviours within a population. This model emphasizes the importance of healthy behaviours being communicated by reliable and trustworthy individuals, who shares similar experiences with the target population. When the communicator is trustworthy, the likelihood adopting a positive health behaviour increase. Furthermore, this approach helps the population to become more willing to accept and integrate a new health behaviour [17]. By using the Diffusion of Innovation model, interventions can utilize opinion leaders or peer mentors to encourage healthy behaviours with relatable experiences, guidance and support. The Diffusion of Innovation model provides a framework for developing interventions to promote healthy behaviour.

### Aim

The aim was to explore Swedish exchange students’ experiences from a semester abroad.

## Method

A qualitative design with semi-structured interviews was used to explore exchange students’ experiences from abroad. The data were analysed using manifest qualitative content analysis [18–20].

### Procedure

The sampling methods were strategic and snowballing. Students at two universities in southern Sweden who had spent parts of their education abroad, were contacted via email and invited to participate in an interview about their experiences from abroad. The student departments supplied the email addresses, and after the interview, the students were asked if they had friends or classmates that had also been abroad on exchange studies. If they had, they were asked to relay the contact information to the first author. The email contained information about the study, its aim, and that participation was voluntary. A form asking for informed consent was also attached and had to be completed before the interview. All students gave oral and written consent. The inclusion criteria for all participants were that they had completed parts of their education abroad at university level, and all students had to be above 18 years of age. Exclusion criteria included students who had only participated in short-term travel or study visits not formally recognized as part of an exchange program, as well as those who had taken part in exchange programs at the high-school level. Students who did not respond to the invitation or declined to participate were excluded from the study; no other exclusion criteria were applied.

### Sample

A total of 11 students were included in the study, out of which four were still enrolled at the university; the rest had graduated. Eight were male and three were female. Two were nursing students, three social work students, one was a tourism major, one an engineer student, and four were economics students. The nursing student and the three social work students had completed between five and 10 weeks of minor field studies, while the others had spent at least one whole semester abroad. One student had spent a semester abroad in 2010, one in 2015, and the rest between 2018 and 2023. Two students had spent time in Peru, two in Spain, the others in Italy, South Africa, New Zealand, South Korea, the US, Philippines, and Palestine. All participants were born, and had grown up, in Sweden. The exchange students were between 23 and 35 years of age when they travelled abroad. All students who consented to participate remained in the study until its completion.

### Data collection

Data were collected using semi-structured interviews; three students were interviewed in 2022, five in 2023, and three in 2024. We used an interview guide, developed for this study based on previous research [12, 15], with open-ended questions to enable the students to share their experiences; the interview guide was piloted in two focus group discussions with other university students (see Supplementary Material). The main author carried out the interviews. The interviews were held digitally in Swedish via Zoom and were recorded and transcribed. Examples of questions include: describe some positive and negative experiences; how did you prepare for your journey?; how was your health affected by a semester abroad?; did you change your behaviour while abroad?; what advice would you give to future exchange students? The interviews lasted between 30 and 60 min. Data saturation was reached after nine interviews, as no new information emerged during the final interviews. This decision was based on repeated readings of the transcripts, analysis, and ongoing discussions within the research team.

### Data analysis

The data were analyzed using a manifest qualitative content analysis [18–20]. A content analysis with a low level of interpretation and abstraction was chosen, as the responses were factual, leaving little room for interpretation, and the data was considered descriptive [18].

**Fig. 1:**
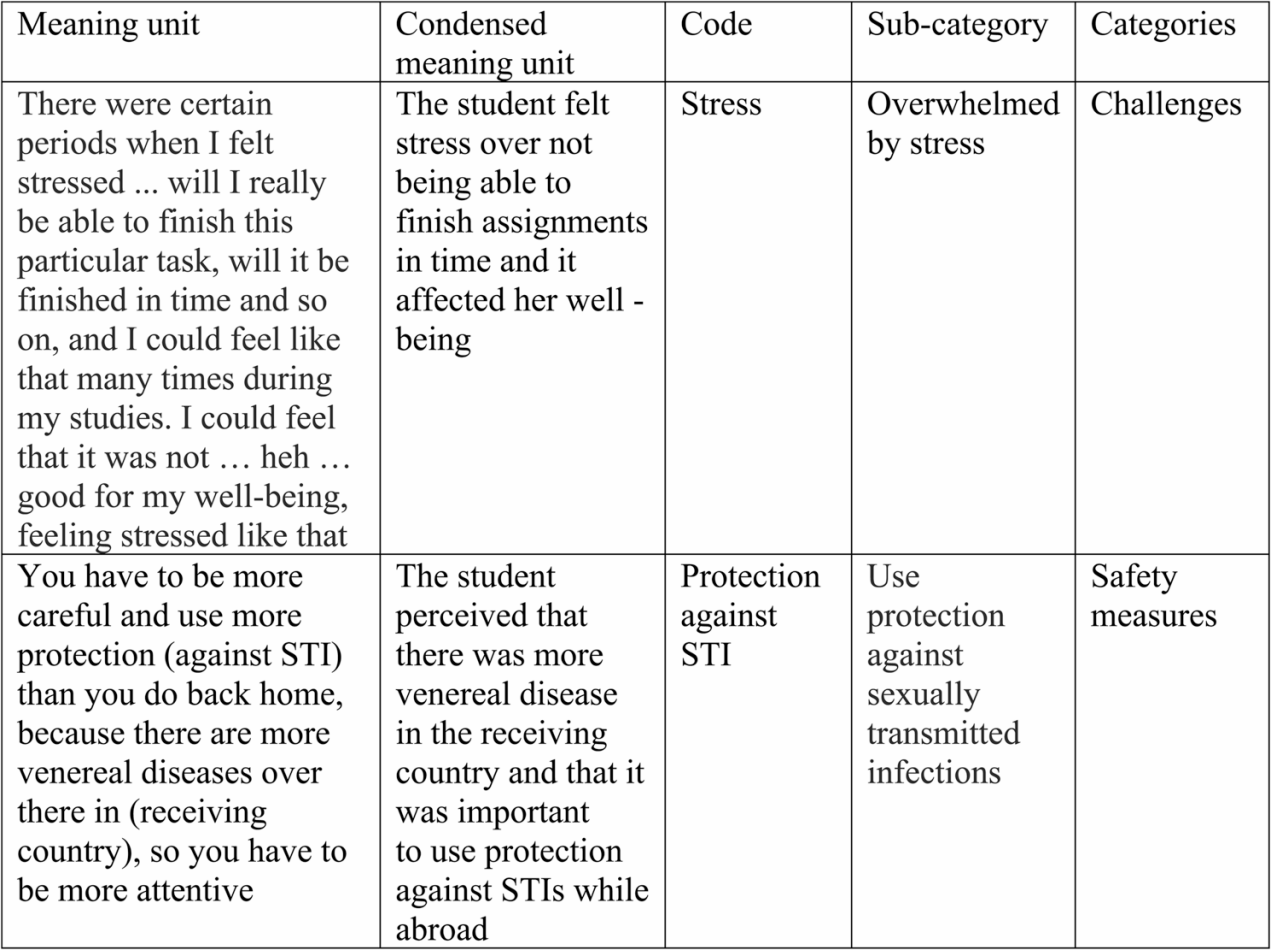
Examples of meaning units, codes, sub-categories and categories

The transcripts were read through multiple times to create an overall understanding of the content. After the initial reading, the data were exported to Microsoft Excel and organized into separate meaning units. The meaning units were then condensed and labelled with a code. The codes were compared based on their similarities and differences, and sorted into eight sub-categories. The sub-categories were then sorted into three categories. Examples of the analysis process in Fig. 1.

## Results

The findings are organized into three categories, based on the experiences of 11 Swedish exchange students who spent at least five weeks abroad: Challenges, Being social, and Safety measures.

### Challenges

#### Overwhelmed by stress

The exchange students had experienced stress during their semester abroad, mainly connected with their studies. They described fear of not being able to hand in assignments on time and failing courses so that they could not graduate on time. It was suggested that since there was limited time to retake a test, it was important not to fail any exams. Some had also experienced stress before departure as there was a lot of preparation that had to be done, such as finding a place to stay, filling out applications for scholarships, and contacting the host university; there was also a fear of not being able to communicate due to not speaking the native language. Others had felt overwhelmed due to being far away from home and the workload. However, in general, the exchange students experienced well-being during their exchange trip. Some had minor medical conditions such as stomach flu, altitude sickness, and urinal tract infections. Loneliness and homesickness were not prominent among the exchange students, with just a few feelings that it was difficult to be away from family and friends for such a long time. Those who had experienced homesickness explained that it was worse in the beginning but wore off after a while.

*“It got really stressful because there was so much that had to be arranged and all that*,* so… then you really felt… there were certain periods when I felt quite stressed… like*,* will I really be able to pull this off… and I mean*,* particular assignments*,* whether I would actually get it done*,* hand it in and so on” Female 3*.

#### Frustrating and fascinating cultural differences

It was mentioned that there could be cultural differences in the receiving country, and that it was important to show respect. The cultural differences were described as both frustrating and fascinating, and most of the students experienced it to some degree. Some students mentioned the importance of accepting that everything is not the same as in your own country, such as people not showing up in time for appointments, or things not being done as thoroughly or properly as back home. Other examples of included the unequal and discriminatory views in some countries, which the exchange students experienced as conservative. These included expectations for women to dress in a certain way, not provocatively, and to avoid drinking alcohol openly. It was described that other people wanted to be social all the time due to the social norms of the receiving country, and there was no time to contemplate alone. The exchange students had different views on preparations before their semester abroad; some recommended reading up on the country’s culture, norms, and possible social activities, while others found it more enjoyable with less preparations to increase the overall experience.

*“Coming from Sweden*,* or Europe in general*,* you’re kind of used to things being structured and orderly. But over there*,* you have to rethink that a bit*,* because things just aren’t handled with the same level of precision. People might not show up at a set time*,* and things aren’t always done the way you’d expect.” Male 1*.

#### A risk of being exposed to violence

It was emphasized that the risks of being exposed to violence could be higher than in the home country. Some of the exchange students had been severely warned by other students, the local population, and staff members about the dangers of being in the country and to be extra careful, and this had startled them. However, most of the students had not been afraid during their exchange trip, and explained that they felt quite safe, calm, and had been well received and welcomed. However, one female student had ended up in an armed conflict and had witnessed people being shot, soldiers carrying weapons, being exposed to tear gas, and feared for her life. A few students described that they had been exposed to theft, one female student had been exposed to sexual harassment in the form of catcalling, and one male student had been robbed on two different occasions by assailants armed with knives.

*“You don’t really feel the same level of safety in Southern Europe. I got robbed twice*,* and some of my friends had their phones stolen*,* stuff like that just happened.” Male 9*.

### Being social

#### Casual drinking

Most of the exchange students had changed their alcohol habits. The female students described that they had been using less alcohol while abroad as they were more isolated from other students, and it varied for the male students. However, binge drinking was not common; instead, the exchange students described a more frequent and social drinking, such as casually and spontaneously going out to bar or pub with other students and having a few drinks, as well as drinking a few glasses of wine while studying or drinking at home with fellow exchange students. It was mentioned that drinking alcohol could be a way of finding friends and to better fit in with other students. The students had not felt pressured to drink alcohol, nor experienced degradation or humiliation at the introduction. A few male students had been partying a couple of times in such a way that it resulted in a hangover, but without any other negative consequences. One male student had participated in an arm-wrestling contest during binge-drinking and hurt his arm, which resulted in a visit to the emergency room and three follow-up hospital visits.

*“Yes*,* it can be more frequent and not just on the weekends*,* if some exchange students or other students tell you that we’re going to this … we’re going to … we’ve found a new pool hall near the university*,* are you coming? Then we would go and have a few beers and it becomes … it becomes more spontaneous …” Male 4*.

#### Casual sex

The female students explained that they did not have sex with new partners during their semester abroad, as they had either visited conservative countries, which made it less likely to find a sexual partner, and/or had boyfriends back home. Some male students commented that they had sex with new partners while abroad, and they shared some of their experiences. Sex during the semester abroad was described as fun, enhanced the experience, and was part of the adventure. It was also described as less emotional than back home, as they knew that the semester would eventually end, and they would have to leave. It was mentioned that being anonymous and having less chance of being recognized by friends made it easier to have casual sex with new people. It was also mentioned that sex while abroad had occurred, but it had not been as frequent as back home, due to a more limited social network which made it more difficult to find a sexual partner. However, some of the male students had met new girlfriends from the local population while abroad and continued the relationship after the semester had ended.

*“Yes*,* so it becomes more like … if you have sex over there*,* it becomes like*,* I don’t really know how to explain it*,* but usually it’s more superficial than if you have sex with someone back home.” Male 10*.

### Safety measures

#### Use protection against sexually transmitted infections

The exchange students were divided in their attitudes towards the risks of having sex while being abroad. Some claimed that there are no differences in having sex while abroad, while others had a more restrictive view. The importance of using protection against STIs was emphasized, as they were in a context where sex with strangers was more common. It was also mentioned that it is important to adhere to the social norms, especially when travelling to conservative countries. It was suggested that exchange students view their newfound freedom as a way of “living out” and “experimenting” with sex, as well as thinking less about the consequences, which they believed could lead to increased risk-taking. It was mentioned that the newfound freedom of being far away from home could lead to increased frequency of sex, and sex with strangers.

*“It’s important to protect yourself (against STI) when meeting new people*,* especially in certain areas where some diseases are more common*,* like HIV*,* for example. In those places*,* it’s especially important to be careful and keep that in mind when traveling. But I think*,* in general*,* it’s important to think about protection (against STI) and all that.” Female 3*.

#### Never walk alone

Several of the students had taken precautions to avoid danger and harm in a way that differed from their behaviour in Sweden, generally being more careful and observant. It was also mentioned that it was important to adhere to social norms, for instance, wearing appropriate clothing to reduce the risk of being harassed. Some students had been afraid of theft and robbery and took extra precautions, such as keeping an extra eye on their personal belongings and keeping their valuables close. Other examples were being extra careful to lock the door when coming home, not going out alone was frequently mentioned, as well as using public transport instead of going alone in a taxi, and not going home alone from a party or after a visit to a pub.

*“It was more like*,* when we were out at the club*,* you’d feel like*,* okay*,* I want to go home now*,* and then someone else would say*,* yeah*,* let’s go home together. Like*,* maybe we shouldn’t walk home alone. So we’d take a taxi*,* even if we couldn’t really afford it*,* you know… there was definitely a different kind of safety awareness there.” Male 5*.

#### Never drink alcohol alone

It was explained that it is easy to adapt to fellow students’ partying and drinking habits, which could lead to over-consumption if not careful. It was also frequently mentioned that excessive alcohol consumption can have negative effects on one’s study results. It was emphasized that it is important to know what kind of alcohol is being consumed, for instance in a proper bar or pub, to avoid homebrewed alcohol, and to never drink alone, only with a group of friends where it is safe, and to be extra careful that no one can slip drugs into one’s glass. Some of the exchange students emphasized the importance of adapting to the local drinking culture. e.g., students who had travelled to conservative countries or remote locations described that they did not use alcohol as much as they did at home as it could be frowned upon.

*“You should be more careful when you drink alcohol while you are travelling*,* for example*,* someone might slip something in your drink if you don’t keep an eye on your glass*,* and always buy alcohol in a proper place*,* such as a bar or a pub.” Female 3*.

#### Meeting new friends

The exchange students strongly recommended a semester abroad and to take the chance, as it might be a once in a lifetime experience and a great opportunity for learning and for meeting new friends. Many of the exchange students had met friends that they still had contact with via social media. They emphasized the importance of enjoying the stay as much as possible, and to not only focus on studies, as there was much that could be learned about the country itself. It was suggested that students should travel, if possible, outside of the campus and get to know the country, its history, and culture, and to get involved in social events, sports, and focus on meeting new friends.

*“You get to know so many new people*,* I think I lived with people with 10 different nationalities and now I have a friend in almost every capital city in Europe. It is fun to develop new friendships*,* it’s cool … So*,* go on an exchange trip*,* that is my best advice.” Male 11*.

## Discussion

Our findings suggest that the exchange students experienced stress due to a high workload and found that cultural differences could be both frustrating and fascinating. Some exchange students experienced minor illnesses and homesickness. The exchange students expressed a responsible view on sex and emphasized using protection against STIs while engaging in casual sex. Some enjoyed drinking alcohol to varying degrees and meeting new friends while abroad. However, they rarely engaged in excessive drinking; instead, they adopted a more spontaneous and social drinking pattern. Some experienced violence, such as robbery and harassment, and most took several safety measures, including being extra careful to lock their doors, not going out alone, and using public transport instead of taxis.

The first category, Challenges, illustrates factors that had an impact on the exchange students’ time living abroad and their well-being. Some had experienced minor illnesses and homesickness; those who had experienced homesickness explained that it was worse at the beginning of the semester but soon wore off, which other exchange students also reported in previous research [21].

Several students had experienced high levels of stress; a high workload and fear of not being able to graduate in time were described as contributing factors, which is in line with previous research [22]. Stress is not unique to students on exchange programmes, stress is commonly reported for Swedish university students in general [23], as is the risk of stress-related mental ill-health [24].

In addition to stress commonly reported among Swedish university students, such as high academic pressure and mental health risks [23, 24], the exchange students in our study described that the fear of not being able to graduate on time, due to failing a course, was intensified by the limited opportunity to retake exams, which created a heightened sense of urgency. This aligns with previous research [22], where it is emphasized that academic pressure is common among exchange students. Moreover, the preparation phase prior to departure, such as securing housing, applying for scholarships, and difficulties with communication, contributed to stress. However, most students reported a general sense of well-being during their semester abroad, indicating that the stress was largely situational and transient.

Another factor was the cultural differences between the home country and the receiving country, which were described as both frustrating and fascinating, but also important to accept. Exchange students in other studies described it as an experience from a different perspective, other than being a tourist, and that it could increase cultural awareness [2, 22, 25].

The second category, being social, involves activities other than studies that the exchange students had participated in. Our findings illustrate that the exchange students enjoyed drinking alcohol to various degrees and meeting new friends while staying abroad. Alcohol consumption became more social, casual, and spontaneous, with less binge-drinking than back home, but exchange students in other studies estimated that they used more alcohol in general while they were abroad [6, 7]. Binge-drinking has also been reported as more common [8]. In addition, the likelihood of a student spending a semester abroad increases if the parents have a high income and are economically well-off [26], and as such, the student can afford to get involved in activities and partying. There might be several reasons why exchange students drink alcohol, and it is open for speculation, but the exchange students in our study suggest that it can be a way of finding new friends and to better fit in. Previous research indicate that excessive alcohol consumption while abroad can lead to poor academic performance [27].

The third category, safety measures, describes precautions that the exchange students had taken to avoid being involved in dangerous situations. There were some concerns among the exchange students that the risk of being exposed to robbery, theft, and violence could be higher. Therefore, they took several safety measures to avoid danger, including being extra careful to lock their doors, not going out alone, and using public transport instead of a taxi.

Previous research on Swedish exchange students shows that there have been instances of sexual violence while abroad [16], however, studies from the US show that exchange students are not at a higher risk of being exposed to sexual violence [28], and female exchange students are less exposed to sexual assault than female campus students [29]. It is, however, difficult to draw definitive conclusions from this, since the study was conducted only in the US and has not been replicated elsewhere.

From our findings, we interpret that the exchange students became aware of the risks associated with being abroad, yet considered them worth taking to some extent. The willingness to take risks has previously been described as essential for exchange students to fully enjoy a semester abroad [2]. Studying abroad has also been described as a confidence booster [22]. Precautions, together with high confidence, might have contributed to the feelings of safety that the exchange students in our study described.

The exchange students in our study also described that having sex with new partners while abroad could be associated with increased risks and emphasized the importance of using protection against STIs. On the other hand, previous research indicates that having unprotected sex with a new partner while abroad is common among Swedish exchange students [15], as well as Swedish youths in general [16]. It is possible that this is a calculated risk-taking. Previous research has also found that exchange students experienced stigma when carrying condoms, as this can be perceived as promiscuous [11].

### Methodological consideration

This study should be read with an understanding of its strengths and limitations. Both the interviewer and the exchange students were fluent in Swedish, which reduced the level of misinterpretations. However, the results were later translated into English, which could have led to a loss of important information. The exchange students in our study had visited nine different countries, which could be both a strength and a limitation. It added diversity to the results, but at the same time, different norms and traditions in the receiving countries could have influenced the exchange students’ behaviour. Only three women participated in the study, which could have caused a misrepresentation in favor of either sex; this could have affected the results [30].

This study is based on a relatively small sample of 11 participants, the interviews did, however, provide rich material, and the students were eager to share their experiences. The sampling procedure was strategic, and the data were collected over an extended period, resulting in variation in participants’ experiences abroad, ranging from approximately five weeks to a full semester. The abbreviated length of the exchange likely restricted the diversity of experiences relative to a full semester, which may have implications for the interpretation of results and the extent to which findings can be transferred to other contexts. Additionally, for some exchange students, a considerable amount of time had passed between their studies abroad and the interviews, potentially leading to memory lapses or distorted recollections [31].

Some of the interview questions could be perceived as sensitive, such as questions about sex, violence, and alcohol, which might have led the students to present themselves in a more positive manner [32]. On the other hand, the exchange students were interviewed digitally, so they could choose a place where they felt safe and comfortable. This safe environment might have made them more inclined to share sensitive information. The interviews were conducted over an extended period. To ensure consistency, all interviews followed the same semi-structured interview guide, and no changes were made to the questions based on earlier interviews. While the interviewer naturally developed greater familiarity with the topic over time, we strived to maintain a neutral and open-ended approach throughout the data collection.

Five researchers with different backgrounds participated in designing this study, and the process was constantly discussed among us to limit pre-understanding. To ensure dependability, the data collection and analysis process were described as detailed as possible [18]. Examples of the analysis were presented in the method section, and quotes were used to illustrate the results, which strengthens both credibility and dependability [18, 19]. All codes were retained until the analysis was finished and only then excluded if they did not fit the aim, which strengthens credibility [20]. To ensure overall trustworthiness, the authors aimed to maintain the same level of interpretation in every category and sub-category [19]. The results may possibly be transferable to exchange students in a similar context in Sweden.

Implications.

Even though the exchange students had taken various precautions and generally reported feeling safe during their stay abroad, some had been exposed to incidents such as robbery, theft, and sexual harassment. In addition, stress and alcohol consumption were frequently mentioned as recurring challenges.

These findings suggest that there are risks associated with being an exchange student that should be communicated to students prior to departure. By addressing these risks proactively, through for example, preparatory workshops, peer-to-peer guidance, and access to mental health resources, students may be better equipped to navigate the complexities of life abroad while maintaining their well-being. For example, by utilizing the Diffusion of Innovation model [17], health-promoting interventions could be developed where former exchange students communicate their experiences of risks and precautions from a semester abroad to students who intend to spend a semester abroad. For example, former exchange students could highlight the need to be careful with alcohol, as it is easy to adapt to fellow students’ partying and drinking habits. They could also emphasize the importance of using protection against STIs, as sexual encounters can become more casual. Furthermore, they could share precautions to take in order to feel safe and avoid being exposed to violence.

In addition, stress should be acknowledged as a significant factor. Former exchange students could share strategies for managing academic pressure, such as time management and seeking support early, especially given the limited opportunities to retake exams. They could also offer advice on how to navigate the stressful preparations before departure, including housing, scholarship applications, and communication with the host university.

Peer-to-peer communication could help students who intend to spend a semester abroad adopt the importance of healthy behaviour, understand the risks, and take necessary precautions. Regular meetings and digital platforms could be used as forums to spread healthy behaviour. However, there is a need for balance between benefits and risks, so as not to discourage exchange studies.

## Conclusion

Swedish exchange students described stress and casual alcohol consumption as common experiences. They emphasized the importance of STI protection as well as safety measures to avoid exposure to violence. Reports of alcohol consumption, casual sex, violence, minor illnesses, and homesickness highlight the challenges faced during exchange studies. These findings indicate potential risks associated with being an exchange student.

## Supplementary Information

Supplementary Material 1.

## Data Availability

To maintain participants’ anonymity, data cannot be openly shared. However, the data is available for verification purposes on reasonable request.
