# Supplementary material for: Being a Swedish university student in a country far away: a qualitative study
Source: BMC Public Health. 2025 Dec 12;26:216. doi: 10.1186/s12889-025-25929-6 (PMC12817536; doi:10.1186/s12889-025-25929-6)
Supplement: Supplementary file 1 — Supplementary Material 1. [file 12889_2025_25929_MOESM1_ESM.docx]

**Interview guide**

Hello my name is …

Thank you for choosing to participate in this study about exchange students' experiences from abroad. The questions are divided into blocks, we start with general questions. Then we go into preparations for the trip, the stay itself, alcohol, attitude to sex during the trip, violence, then we finish with the return home.

**General questions**

Gender:

Age:

Destination:

Time abroad:

Faculty:

Where did you travel?

Why did you choose to travel?

Tell us more about your exchange trip

**Preparations**

How did you prepare for your journey?

What support/information did you receive?

What support/information would you have liked?

**The stay**

Describe some positive and negative experiences

What would the best exchange trip look like?

Tell me about the introduction to the student life

How was your health affected by a semester abroad?

**Alcohol**

What do you think about alcohol in general?

What is your attitude towards alcohol during an exchange trip?

Is there a difference between drinking alcohol away versus at home?

In which contexts did you drink alcohol?

Did you ever drink too much alcohol?

Is there a relationship between alcohol and study results?

**Sex**

What do you think about sex in general?

What is your attitude towards sex during an exchange trip?

What is the difference between sex away versus at home?

**Violence**

What you think about violence?

What do you think about the risk of being exposed to violence away versus at home

Did you ever feel threatened?

Did you get scared while on your exchange trip?

Were you exposed to violence while abroad?

(did you seek help or did you tell anyone?)

Did you change your behaviour while abroad to avoid being exposed to violence?

Do you know someone who got hurt during their exchange trip?

**The homecoming**

Were you offered any follow-up when you got home?

(If offered, what did you participate in?)

What kind of follow-up would you have liked?

What advice would you give to prospective exchange students?

If the participants gave a yes/no answer, relevant follow-up questions were asked, such as “describe how” and “tell me more”.
